# A Review on Mitotane: A Target Therapy in Adrenocortical Carcinoma

**DOI:** 10.3390/cancers16234061

**Published:** 2024-12-04

**Authors:** Fabiano Flauto, Maria Cristina De Martino, Chiara Vitiello, Rosario Pivonello, Annamaria Colao, Vincenzo Damiano

**Affiliations:** Department of Clinical Medicine and Surgery, University of Naples Federico II, 80131 Naples, Italy; fabianoflauto96@gmail.com (F.F.); demartino.mc@gmail.com (M.C.D.M.); chiara.vitiello@unina.it (C.V.); rosario.pivonello@unina.it (R.P.); colao@unina.it (A.C.)

**Keywords:** adrenocortical carcinoma, mitotane, rare cancers, drug resistance, target therapy

## Abstract

Adrenocortical carcinomas (ACCs) are rare and aggressive malignancies of adrenal cortex, associated with largely unknown mechanisms of biological development and poor prognosis. Currently, mitotane is the sole approved drug for treating advanced adrenocortical carcinomas (ACCs) and is being utilized more frequently as postoperative adjuvant therapy. Although it is understood that mitotane targets the adrenal cortex and disrupts steroid production, its precise mechanism of action requires further exploration. Additionally, mitotane affects cytochrome P450 enzymes, causes the depolarization of mitochondrial membranes, and leads to an accumulation of free cholesterol, ultimately resulting in cell death. Many patients treated with mitotane develop disease progression over time, underlying the need to understand the mechanisms of primary and acquired resistance. In this manuscript, we provide an overview on the intracellular mechanisms of action of mitotane, exploring data regarding predictive factors of response and evidence associated with the development of primary and acquired resistance mechanisms. In this discussion, mitotane is considered a real target therapy.

## Simple Summary

Mitotane is the only reimbursed drug currently approved for adrenocortical carcinoma (ACC), a rare and aggressive malignancy. Despite its clinical use spanning over six decades, the precise mechanisms of mitotane’s adrenolytic action remain poorly understood, and the factors driving resistance to this therapy are elusive. This review delves into the molecular pathways that underpin mitotane’s therapeutic effects, emphasizing its potential as a targeted treatment for ACC. Additionally, it explores the mechanisms of resistance and identifies potential biomarkers that could inform treatment strategies. Drawing on a comprehensive analysis of the scientific literature, the review offers hypotheses regarding these processes while acknowledging the lack of definitive conclusions. Through this, it aims to contribute to a deeper understanding of mitotane’s efficacy and guide future research in overcoming therapeutic resistance in ACC.

## 1. Introduction

Adrenocortical carcinomas (ACCs) are uncommon and aggressive tumors that develop in the adrenal cortex, with an incidence of 0.7–2.0 cases per million people each year [1]. The prognosis for ACCs varies widely, with the primary factors being the tumor’s initial stage, whether surgical resection is possible, and the Ki67 proliferation index, as outlined by the European Network for the Study of Adrenal Tumors (ENSAT) classification [1]. While ACCs can be associated with rare genetic syndromes, such as Beckwith–Wiedemann syndrome (due to alterations on chromosome 11p15.5), Li–Fraumeni syndrome (related to *TP53* mutations), multiple endocrine neoplasia (MEN) type 1, and familial adenomatous polyposis (FAP), most cases are sporadic [1,2,3]. ACCs can occur at any age, with a peak incidence between 40 and 50 years, and they are more common in women, who represent 55–60% of cases. The median overall survival (OS) for patients with ACC is approximately 3–4 years. Five-year survival rates vary: 60–80% for tumors confined to the adrenal gland, 35–50% for locally advanced cases, and significantly lower for metastatic disease, with survival rates reported between 0% and 28% [3]. About 50–60% of ACC cases present with symptoms of hormone excess, typically glucocorticoid, in the case of secretory tumors or otherwise with large tumors which can manifest through mass effect. The current recommendation is to perform a complete en bloc resection of all adrenal tumors suspected to be ACC, including the peritumoral/periadrenal retroperitoneal fat. If adjacent organs are suspected to be invaded, en bloc resection is recommended [3,4,5,6,7]. The aim of the operation is to ensure the local control of disease by achieving negative resection margins (R0). The use of adjuvant therapy for ACC has been a topic of debate but can be appropriate for certain patients, particularly those with positive resection margins or following the resection of localized recurrences [8,9]. ACC prognosis depends on staging, surgical margins, hormonal status, and tumor biology. Advanced stages (III/IV) and positive surgical margins (R1/R2) predict poorer survival [1,10,11]. The Ki67 index is a key marker, with higher values indicating worse outcomes, and functional tumors worsen prognosis [10,11,12,13,14,15,16,17]. Older age and genetic mutations (e.g., *TP53*) are linked to poorer outcomes [18,19,20,21]. Prognostic tools like the Helsinki and modified S-GRAS scores aid risk stratification [10,22,23]. Currently, adjuvant mitotane is the most recommended therapy for ACC according to clinical guidelines. However, emerging evidence from the ADIUVO trial [24] indicates that mitotane may not be necessary for low-risk patients (ENSAT stage I, II, or R0 resection with Ki67 < 10%). An ongoing study, ADIUVO-2, is investigating the effectiveness of mitotane alone compared to mitotane combined with chemotherapy in high-risk ACC patients [25]. For recurrent or advanced ACC, the combination of etoposide, doxorubicin, cisplatin, and mitotane (EDP-M) is recommended as the first-line treatment, according to the FIRM-ACT trial results [26]. This trial showed that the EDP-M regimen is more effective than the streptozocin/mitotane (STZ-M) regimen in terms of recurrence-free survival, although it did not improve overall survival. In second-line treatment, options such as gemcitabine plus capecitabine or STZ, alongside ongoing mitotane therapy, may be considered [27,28]. For patients with a low tumor burden or poor performance status, mitotane can be used as a monotherapy [29]. Mitotane represents the therapeutic backbone for the management of ACC; however, its toxicity profile and lack of predictive markers of response make its use complex and worthy of close monitoring. Table 1 presents all the main studies concerning the use of mitotane.

## 2. Adrenal Gland Steroid Pathways

The adrenal cortex is responsible for producing steroid hormones, such as cortisol, aldosterone, and androgens. The process of steroidogenesis requires cholesterol as a precursor. Therefore, maintaining a constant supply of cholesterol is crucial for proper adrenal function [40]. Adrenocortical cells can obtain cholesterol through several pathways: (1) intracellular de novo synthesis, a process that involves the synthesis of cholesterol within the cell itself from acetyl-CoA, and enzymes such as HMG-CoA reductase play a key role in this pathway; (2) the uptake of circulating high-density lipoprotein (HDL) via scavenger receptors such as SR-B1 (scavenger receptor class B type 1); (3) the uptake of circulating low-density lipoprotein (LDL) through receptor-mediated endocytosis involving LDL receptors, and once inside the cell, LDL is degraded to release free cholesterol; and (4) the mobilization of stored intracellular cholesteryl esters (CEs) within lipid droplets (LDs). When needed, hormone-sensitive lipase (HSL) converts these CEs into free cholesterol, which is then available for steroidogenesis [41]. Disruptions in these cholesterol pathways can significantly affect adrenal function. For example, genetic mutations affecting enzymes involved in cholesterol synthesis or uptake can lead to disorders such as congenital adrenal hyperplasia (CAH). Pharmacological interventions that alter cholesterol metabolism can also impact adrenal health and function [42]. Under normal conditions, adrenocortical cells primarily focus on storing and releasing cholesterol. However, during acute stress, these cells rapidly mobilize cholesterol from LD stores to mitochondria for the synthesis of steroid hormones. This rapid mobilization is essential for an immediate response to stress. Lipid droplets (LDs) are not merely inert storage organelles but are actively involved in managing intracellular lipid and cholesterol flux [43]. This dynamic role is mediated by proteins associated with LDs, such as perilipins (PLINs), CIDEC, and CGI-58 [44]. These proteins interact with lipids, cholesteryl esters (CEs), triacylglycerol (TAG), and enzymes like HSL and adipose triglyceride lipase (ATGL) to regulate lipid storage and mobilization. In adrenocortical cells, lipid droplets primarily store cholesteryl esters. Lipolysis, the breakdown of lipids, is mediated by HSL and ATGL. HSL primarily hydrolyzes cholesteryl esters, while ATGL targets triacylglycerol. The coordinated action of these enzymes ensures a regulated supply of free cholesterol for steroidogenesis. Mitotane impacts lipid and cholesterol metabolism in complex ways [45]. Understanding these interactions is crucial for optimizing mitotane therapy and managing its side effects.

## 3. In Vitro ACC Models for Anti-Cancer Drugs

Researchers commonly use cell models derived from H295 and SW13 cells to study anti-cancer drugs in vitro [46]. The H295 cell line originates from a tumor extracted from a female patient with ACC. These cells, referred to as NCI-H295, act as pluripotent adrenal cells capable of synthesizing zone-specific steroids. However, the original H295 cells exhibit a poorly adherent phenotype and a relatively slow doubling time. To enhance their utility, researchers optimized culture conditions and used commercial sera, leading to the development of three H295R sub-strains [46]. These adaptations have made them more suitable for experimental purposes. Another variant, H295A, was produced by removing non-adherent cells during passaging, resulting in a strain that responds to angiotensin II. The parental H295 cells are more glucocorticoid-producing, H295R cells primarily produce androgens, and H295A cells are more inclined toward mineralocorticoid production. To create a novel human adrenocortical carcinoma (HAC) cell line responsive to ACTH, Parmar et al. [47] isolated clonal populations from what was initially believed to be a new adrenal tumor. However, single-nucleotide polymorphism (SNP) array analysis later revealed that these clones were contaminants from H295R cells. Among the isolated clones, HAC13 and HAC15 showed significant aldosterone production in response to angiotensin II (Ang II) and potassium (K^+^), while HAC15 also demonstrated modest cortisol production and increased steroidogenic enzyme expression in response to ACTH. Unlike NCI-H295 strains, which originate from a mixed tumor cell population, HAC cell clones are monoclonal, potentially offering greater stability in steroid production over extended cultures [48]. Another cell model often employed in mitotane studies is the SW13 cell line, derived from a 55-year-old woman with small-cell carcinoma of the adrenal cortex. Notably, SW13 cells lack steroidogenic capability, and their origins, whether primary ACC cells or metastatic cells from another tumor, remain unclear. Nevertheless, their resistance to mitotane makes them a useful model for research [49]. To broaden the range of ACC models, scientists have also developed protocols to establish cell lines from patient-derived tumor xenografts (PDTXs). These models preserve the original tumor characteristics but often have limited growth capacity. For instance, the first adult ACC PDTX and its corresponding cell line, MUC-1, were derived from a 24-year-old male patient with a supraclavicular ACC metastasis [49,50]. MUC-1 cells retain the hormonal activity and phenotypic traits of ACC even after several passages and exhibit resistance to standard drug treatments, including mitotane. Using a similar approach, Kiseljak-Vassiliades et al. generated two additional ACC cell models: CU-ACC1 and CU-ACC2 [51]. CU-ACC1 was established from a 66-year-old hypertensive patient with hypokalemia and carries a *CTNNB1* mutation in exon 3. CU-ACC2, derived from liver metastases of a 26-year-old patient with Lynch syndrome, has a deletion in exons 1–6 of the MSH2 gene, characteristic of the syndrome [51]. These models, particularly those derived from PDTXs, provide valuable tools for investigating ACC, evaluating anti-cancer drugs like mitotane, and uncovering mechanisms of drug resistance and potential therapeutic targets [49]. Patient-derived tumor organoids (PTOs) are three-dimensional laboratory-grown cell structures created using samples taken directly from patients. These in vitro models are tailored to the individual and offer significant advantages over conventional two-dimensional cell cultures, established cell lines, and animal models. This is because they closely mimic the original human tumor’s characteristics and provide personalized insights for large-scale drug testing. Specifically, PTOs developed from ACC serve as a robust tool for investigating cancer-related signaling pathways and devising customized treatment strategies [49,52].

## 4. Mitotane Pharmacokinetics and Pharmacodynamics

**Figure 1:**
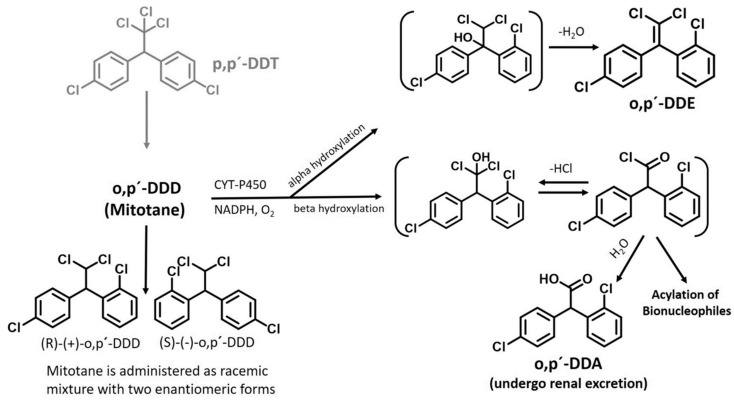
Mitotane Metabolism. p,p′-DDT: p,p′-dichlorodiphenyl-trichloroethane; o,p′-DDD: o,p′-dichlorodiphenyl-dichloroethane; p′-DDE: o,p′-dichlorodiphenyl-dichloroethene; o,p′-DDA: o,p′-dichlorodiphenyl-acetic acid. This schematic illustrates the metabolic pathways of mitotane and its various metabolites, highlighting the transformations that occur within the body. (This diagram was created with BioRender.com.)

Mitotane, also known as 1,1-(o,p′-Dichlorodiphenyl)-2,2-dichloroethane (o,p′-DDD) and marketed as Lysodren^®^ (HRA Pharma Rare Diseases, Paris, France), is a compound derived from the insecticide DDT. Once inside adrenal cells, it undergoes mitochondrial metabolism, transforming into two metabolites, namely DDE (1,1-(o,p′-Dichlorodiphenyl)-2,2-dichloroethene) and DDA (1,1-(o,p′-Dichlorodiphenyl) acetic acid), through processes known as α-hydroxylation and β-hydroxylation, respectively [53,54,55] (Figure 1). An intermediate metabolite, o,p′-dichlorodiphenyl acyl chloride (DDAC), is produced by cytochrome P450 enzymes (CYP450). This acyl chloride is highly reactive and can interact with specific cellular nucleophiles, inducing an adrenolytic effect through oxygen activation [55]. Though not fully proven, it is widely believed that oxidative damage and free radical production are central to the cytotoxicity of mitotane. The acyl chloride primarily binds to proteins associated with phospholipids, and when water is added, it forms o,p′-DDA, which is excreted in the urine. Recent studies suggest that o,p′-DDA itself is inactive, showing no anti-tumor effects in adrenal cells and not inducing oxidative stress or apoptosis [56]. DDAC, on the other hand, can either bind to mitochondrial macromolecules in adrenal cells or be further metabolized by CYP2B6 in the liver or intestines, potentially reducing mitotane’s bioavailability. Due to its lipophilic nature, mitotane accumulates in adipose tissue, creating a depot effect that leads to its gradual release into the bloodstream, thus contributing to its prolonged half-life and sustained therapeutic effects [54]. This slow release helps maintain steady mitotane levels in the blood, which is essential for treating ACC. The therapeutic range for mitotane is typically between 14 and 20 mg/L (about 40–60 µM), and it is important to keep levels within this range to achieve an anti-tumor effect while minimizing the risk of severe neurological toxicity [57,58]. Retrospective studies have shown that mitotane concentrations of ≥14 mg/L are associated with favorable disease responses in both advanced and adjuvant ACC treatment [36]. However, levels above 20 mg/L are linked to a higher risk of central neurological toxicity, though mild symptoms can occur at lower concentrations. Interestingly, some evidence suggests that steroid secretion inhibition may still occur at lower mitotane levels, indicating potential therapeutic benefits even outside the standard therapeutic window [59].

### 4.1. Impact on Mitochondrial Enzymes and Steroidogenesis Disruption

Mitotane exerts its primary effects on the adrenal cortex, leading to cellular destruction and impaired steroidogenesis. While it also affects the gonads and pituitary gland, its predominant action is observed in the adrenal cortex, specifically targeting the zona fasciculata and zona reticularis, with minimal impact on the zona glomerulosa [60]. This specificity results in less pronounced effects on aldosterone secretion. Mitotane induces cellular toxicity in a dose-dependent manner by rupturing mitochondrial membranes within adrenal cells [60,61]. The high concentration of enzymes involved in steroidogenesis and cholesterol metabolism in these cells likely contributes to mitotane’s selective action. Mitotane affects cholesterol transport and steroidogenesis initiation, through significantly decreasing the expression of the Steroidogenic Acute Regulatory Protein (STAR), which is crucial for the transport of cholesterol into the mitochondria. The STAR facilitates the first step in steroidogenesis by moving cholesterol to the inner mitochondrial membrane, where it is converted into pregnenolone by the enzyme cholesterol side-chain cleavage enzyme, P450scc (CYP11A1) [61]. Mitotane not only reduces the transcription of the STAR gene but also inhibits the functional activity of CYP11A1, thereby impairing the initial step of steroidogenesis and reducing the synthesis of pregnenolone, the precursor for all steroid hormones. Mitotane downregulates several other key steroidogenic enzymes such as 3β-hydroxysteroid dehydrogenase/Δ5-4 isomerase (HSD3B2), an enzyme essential for the biosynthesis of all classes of steroid hormones, and steroid 21-hydroxylase (CYP21A2), which is critical for the biosynthesis of glucocorticoids and mineralocorticoids. The downregulation of CYP21A2 affects the production of cortisol and aldosterone. One of the most important targets of mitotane is 11β-hydroxylase (CYP11B1) [62]. This enzyme converts 11-deoxycorticosterone and 11-deoxycortisol into corticosterone and cortisol, respectively. The impact of mitotane on CYP11B1 is complex and varies depending on the experimental conditions and cell lines used. Some studies have reported downregulation of CYP11B1, while others have observed upregulation. Despite this variability, mitotane interacts with CYP11B1, creating an irreversible bond that decreases both cortisol and aldosterone secretion in a concentration-dependent manner. However, the inhibition of CYP11B1 by mitotane does not appear to be essential for its overall cytotoxic effect, as evidenced by the fact that cells lacking CYP11B1 are still susceptible to mitotane. Mitotane also inhibits aldosterone synthase (CYP11B2), responsible for the final steps of aldosterone synthesis. Mitotane inhibits the transcription of the *CYP11B2* gene, reducing aldosterone production. All these enzyme inhibitions, mediated by mitotane, generate mitochondrial dysfunction [61]. Mitochondria are crucial organelles involved in the energy production, metabolism, and the regulation of cell death pathways, including apoptosis. The dysfunction caused by mitotane manifests in several ways [45,61]. (1) Disruption of membrane potential: Mitotane impairs the mitochondrial membrane potential, which is necessary for maintaining mitochondrial function and ATP production. This loss of membrane potential results in bioenergetic failure and cell death. (2) Increased ROS production: Mitotane increases reactive oxygen species (ROS), such as superoxide radicals and hydrogen peroxide, leading to oxidative stress and damage to cellular components, including DNA, proteins, and lipids. Excessive ROS production can further damage mitochondria and trigger apoptosis. (3) Disruption of respiratory chain activity: Mitotane inhibits cytochrome c oxidase (complex IV) activity in a dose-dependent manner in H295R cells, and it also reduces the activity of Complex I (NADH–ubiquinone oxidoreductase). However, it has little impact on Complex III (ubiquinol–cytochrome c oxidoreductase) and Complex II (Succinate dehydrogenase) [45]. (4) Release of pro-apoptotic factors: The mitochondrial dysfunction caused by mitotane can result in the release of pro-apoptotic factors like cytochrome c into the cytoplasm, triggering caspase activation and leading to cell death.

Mitotane also affects the steroidogenic factor 1 (SF-1), a nuclear factor crucial for expressing P450 steroidogenic enzymes in the adrenal glands and gonads [60,61]. SF-1 acts as a transcription factor that binds to specific DNA sequences in the promoter regions of target genes, enhancing their transcription. This regulation is crucial for the expression of steroidogenic enzymes, including CYP11A1, CYP11B1, and other cytochrome P450 enzymes involved in steroid biosynthesis. SF-1 activity is modulated by cyclic AMP (cAMP), a second messenger that is elevated in response to adrenocorticotropic hormone (ACTH) stimulation. ACTH binds to its receptor on adrenal cells, activating adenylate cyclase, which converts ATP to cAMP. Increased cAMP levels activate protein kinase A (PKA), which phosphorylates SF-1 and enhances its transcriptional activity. SF-1 functions in concert with various co-regulators, including co-activators and co-repressors, to fine-tune the expression of target genes [60,61]. This interaction ensures precise control over steroid hormone production. Mitotane interferes with the ACTH/cAMP signaling pathway, which is essential for SF-1 activation. By inhibiting this pathway, mitotane reduces the phosphorylation and activation of SF-1, thereby decreasing the expression of genes such as *STAR*, *CYP11A*, *CYP11B1*, and *CYP11B2*, which are critical for steroidogenesis. The effects of mitotane on ACTH/cAMP signaling and SF-1 activity can vary depending on the cell model used. For instance, H295R adrenal carcinoma cells show variable responses to ACTH depending on subclone and culture conditions, while H295A cells are generally non-responsive to this hormone [62]. SF-1 regulates the expression of genes involved in cholesterol transport, such as *ABCA1* and *SCARB1* (which encodes for scavenger receptor BI, SR-BI). Mitotane reduces the expression of these genes, leading to impaired cholesterol efflux and uptake, which is critical for steroidogenesis. This reduction in cholesterol transport contributes to the accumulation of intracellular cholesterol and further disrupts steroid hormone synthesis. While SF-1 plays a role in maintaining lipid homeostasis, mitotane induces lipid peroxidation without triggering ferroptosis, a form of iron-dependent cell death associated with increased lipid peroxidation [60,62]. This selective disruption highlights the complex role of SF-1 in lipid metabolism and cellular resistance mechanisms. The increased intracellular free cholesterol and oxysterols, such as 27-hydroxycholesterol, could modulate SF-1 activity and affect cellular responses to mitotane. Given SF-1’s role in regulating cholesterol homeostasis and steroidogenesis, targeting pathways that interact with SF-1, such as LXRα and PCSK9, could enhance mitotane’s efficacy. These approaches aim to modulate cholesterol levels and metabolic stress, potentially reducing mitotane resistance and optimizing therapeutic outcomes [63]. Another critical target of mitotane pharmacodynamics is Sterol O-Acyltransferase 1 (SOAT1), also known as Acyl-CoA: cholesterol acyltransferase 1 (ACAT1) [64,65]. SOAT1 is an enzyme located in the endoplasmic reticulum (ER) and mitochondria-associated membranes (MAMs). It catalyzes the formation of cholesteryl esters from free cholesterol and fatty acyl-CoA. This process is essential for maintaining cellular cholesterol homeostasis by converting potentially toxic free cholesterol into less harmful storage forms. By esterifying free cholesterol, SOAT1 helps regulate lipid metabolism and prevent the accumulation of free cholesterol in cell membranes, which can lead to cellular dysfunction and apoptosis [63]. Research has identified SOAT1 as a key molecular target of mitotane. The inhibition of SOAT1 by mitotane disrupts cholesterol homeostasis in adrenal cells, leading to a cascade of cellular effects that contribute to its cytotoxicity. Mitotane inhibits SOAT1 activity, leading to the accumulation of free cholesterol within the cell [64]. Excessive free cholesterol can integrate into cellular membranes, causing increased membrane rigidity and dysfunction. The buildup of free cholesterol in the ER triggers ER stress, which activates the unfolded protein response (UPR). Prolonged ER stress can lead to apoptosis, contributing to mitotane’s cytotoxic effects on ACC cells. In addition to inducing ER stress, the accumulation of free cholesterol in mitochondria can disrupt mitochondrial function. This includes depolarization of the mitochondrial membrane, impaired ATP production, and increased ROS production, all of which contribute to cell death. The disruption of ER and mitochondrial function leads to the release of pro-apoptotic factors such as cytochrome c from mitochondria into the cytoplasm [60,61,62,63,64,65]. This release triggers the activation of the caspase cascade, culminating in apoptotic cell death. A summary of mitotane targets is given in Table 2.

### 4.2. Effects on Mitochondria-Associated Membranes

Mitochondria-associated membranes (MAMs) are specialized, dynamic connections between the mitochondria and the endoplasmic reticulum (ER) that play a crucial role in transporting specific lipids, such as cholesterol, into the mitochondria. When H295R cells are treated with mitotane, significant changes occur in mitochondrial structure, including a decrease in the number of intact mitochondria [68]. This is often accompanied by a more fragmented, punctate appearance. Additionally, mitotane disrupts the integrity of MAMs, reducing the interactions between the mitochondria and the ER. These alterations may lead to the gradual depolarization of the mitochondrial membrane, possibly caused by the inhibition of cytochrome c oxidase (COX) enzymes, which disrupt the mitochondrial respiratory chain and contribute to the breakdown of MAMs. Sterol O-acyltransferase enzymes (SOAT1 and SOAT2), which are found in MAMs, catalyze the conversion of free cholesterol and acyl-CoA into cholesteryl esters. The inhibition of SOAT activity by mitotane is believed to increase free cholesterol levels, leading to ER stress and apoptosis. Studies have identified SOAT1 as a key target of mitotane, with evidence suggesting that SOAT1 expression correlates with the effectiveness of adjuvant mitotane treatment [64]. However, more recent studies have not consistently confirmed SOAT1 as a reliable predictor of mitotane treatment response [65].

**Figure 2:**
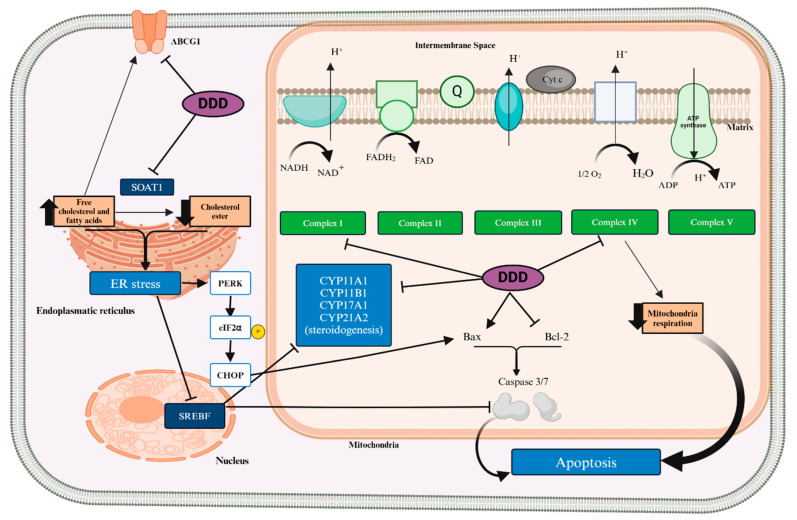
Mechanism of the Anti-Tumor and Anti-Secretory Effects of Mitotane (DDD) on Adrenal Cells. This figure illustrates the underlying mechanisms by which mitotane exerts its anti-tumor and anti-secretory effects on adrenal cells. Key components include: ABCG1: ATP-binding cassette subfamily G member 1; ADP: Adenosine diphosphate; ATP: Adenosine triphosphate; Bax: BCL2-associated X apoptosis regulator; Bcl-2: B-cell lymphoma protein 2; CHOP: CCAAT-enhancer-binding protein homologous protein; Complex I: Ubiquinone oxidoreductase; Complex II: Succinate dehydrogenase; Complex III: Ubiquinol—cytochrome c oxidoreductase; Complex IV: Cytochrome c oxidase; Complex V: ATP synthase; CYP11A1: Cytochrome P450 family 11 subfamily A member 1; CYP17A1: Cytochrome P450 family 17 subfamily A member 1; CYP21A2: Cytochrome P450 family 21 subfamily A member 2; CYP11B1: Cytochrome P450 family 11 subfamily B member 1; Cyt c: Cytochrome c; ER: Endoplasmic reticulum; eIF2α: Eukaryotic initiation factor 2 α phosphorylation; FAD: Flavin adenine dinucleotide; NADH: Nicotinamide adenine dinucleotide; PERK: Eukaryotic translation initiation factor 2 α kinase 3; SOAT1: Sterol O-acyltransferase 1; SREBF: Sterol regulatory element-binding transcription factor 1; Symbols:→: Activation; ┤: Inhibition. (This figure was created with BioRender.com.)

In vitro studies show that mitotane induces ER stress by inhibiting SOAT1, which disrupts cholesterol and steroid hormone synthesis. The buildup of free cholesterol becomes toxic to cells. MAMs are essential for various cellular functions, including lipid metabolism, calcium signaling, and apoptosis. Mitotane’s effects on MAMs can disturb cellular balance and contribute to its toxic effects: (1) Lipid Metabolism: Mitotane may interfere with lipid metabolism at MAMs, disrupting the transfer and synthesis of lipids between the ER and mitochondria. This disruption can compromise cell membrane integrity and function, leading to cell death. (2) Calcium Signaling: MAMs are critical for calcium ion transfer between the ER and mitochondria, regulating cellular calcium levels and signaling. Mitotane-induced changes to MAM function can alter calcium dynamics, triggering mitochondrial dysfunction and promoting apoptosis. (3) Apoptotic Signaling: MAMs are involved in coordinating the release of pro-apoptotic factors and in responding to ER stress signals. The disruption of MAM integrity and function by mitotane can enhance apoptotic signaling, ultimately driving cell death in ACC cells [64,65,66,67,68] (Figure 2).

### 4.3. Effects on Cytoskeleton

Mitotane has been reported to disrupt the dynamic assembly and disassembly of microtubules, which are essential for various cellular processes, including cell division, intracellular transport, and cell morphology. Microtubules are composed of tubulin protein subunits and undergo dynamic instability, with periods of growth (polymerization) and shrinkage (depolymerization) [74]. Mitotane treatment may interfere with microtubule polymerization and stability, leading to aberrant microtubule structures and impaired cellular functions dependent on intact microtubules. As microtubules play a critical role in mitotic spindle formation and chromosome segregation during cell division, the disruption of microtubule dynamics by mitotane can lead to mitotic spindle defects and cell cycle arrest in G2 phase [62]. The mitotane-induced disruption of the mitotic spindle may prevent proper chromosome alignment and segregation, resulting in mitotic catastrophe and cell death [75].

### 4.4. Protein Synthesis Inhibition and Induction of Protein Abnormalities

Mitotane has been reported to inhibit protein synthesis in ACC cells. Although the exact mechanisms underlying this inhibition are not fully understood, protein synthesis is a fundamental process required for cell growth, proliferation, and survival [76]. In addition to its effects on protein synthesis, mitotane treatment also influences protein expression and function in ACC cells [77]. Proteomic studies investigating the effects of mitotane on protein expression profiles in ACC cells have identified changes in the abundance of specific proteins involved in various cellular processes, including cell cycle regulation, apoptosis, and metabolism [78]. Mitotane treatment may alter the expression of key cell cycle proteins, such as cyclins, cyclin-dependent kinases (CDKs), and cell cycle inhibitors. Mitotane-induced alterations in protein expression may modulate apoptotic signaling pathways, promoting programmed cell death in ACC cells.

### 4.5. Induction of Apoptosis

Mitochondria play a central role in regulating apoptotic pathways by releasing pro-apoptotic factors such as cytochrome c into the cytoplasm. Mitotane-induced mitochondrial dysfunction results in the permeabilization of the mitochondrial outer membrane, allowing the release of cytochrome c into the cytoplasm [45,53]. Cytochrome c release activates the caspase cascade, a series of proteolytic enzymes that orchestrate the apoptotic process. Mitotane treatment leads to the activation of initiator caspases, such as caspase-9, which in turn activate downstream executioner caspases, including caspase-3. Activated caspases cleave key cellular proteins, leading to morphological and biochemical changes in the characteristic of apoptosis, such as DNA fragmentation, chromatin condensation, and cell membrane blebbing. Mitotane modulates the expression and activity of Bcl-2 family proteins, which regulate mitochondrial outer membrane permeabilization and apoptosis. The Bcl-2 family includes both pro-apoptotic (e.g., *Bax*, *Bak*) and anti-apoptotic (e.g., *Bcl-2*, *Bcl-xL*) members. Mitotane-induced DNA damage may activate the tumor suppressor protein p53, a key regulator of apoptosis. The activation of p53 leads to the transcriptional activation of pro-apoptotic genes, such as *Bax* and *Puma*, and the inhibition of anti-apoptotic genes, promoting apoptosis. Additionally, p53 can induce cell cycle arrest and senescence in response to DNA damage, providing additional mechanisms to inhibit tumor growth and progression. Mitotane-induced apoptosis may involve crosstalk with other signaling pathways implicated in cell survival and proliferation. Mitotane treatment can modulate signaling pathways such as the PI3K/Akt and MAPK/ERK pathways, which play roles in cell survival and apoptosis resistance [75].

## 5. Predictive Factors of Mitotane Response

Currently, plasma mitotane concentration is the primary predictive biomarker utilized in clinical settings [36,58]. Evidence from clinical studies suggests that achieving high plasma mitotane levels, commonly defined as ≥14 mg/L, is necessary for therapeutic efficacy. This target level is outlined in the prescribing information provided by the US Food and Drug Administration (FDA) and is frequently used as a benchmark in prospective clinical trials. Despite this, many patients undergoing mitotane treatment fail to attain these plasma concentrations, leading to disappointment when lab results show lower levels. Because ACC is a rare condition, all studies on mitotane are retrospective in nature, which introduces various biases such as time-to-event and selection biases [59]. Interestingly, some studies have documented anti-tumor effects in patients with plasma mitotane concentrations below 14 mg/L, though these cases are often disregarded as false positives due to biases and a rigid adherence to the established threshold. Notably, therapeutic responses have been observed even in patients with serum mitotane levels under 14 mg/L, and in some instances, below 10 mg/L. Additionally, the Ki-67 index, a widely recognized marker of tumor aggressiveness in ACC, is often used to predict the disease’s progression and the potential effectiveness of mitotane therapy [13]. While elevated Ki-67 levels typically indicate a poorer prognosis, they may also suggest a greater likelihood of response to mitotane when therapeutic concentrations are achieved.

## 6. Emerging Biomarkers of Response and Resistance

Mitotane remains the primary treatment for ACC, but resistance, both primary and acquired, limits its efficacy [20]. Primary resistance arises from genetic and epigenetic alterations (e.g., TP53, CTNNB1 mutations, gene hypermethylation), the overexpression of drug efflux pumps (e.g., ABCB1), and disruptions in steroidogenic pathways [79,80,81]. Acquired resistance develops during treatment, involving pathways like Wnt/β-catenin, PI3K/AKT/mTOR, and genes such as BCL-2 and TP53 [82]. Understanding these mechanisms is essential for developing targeted therapies and improving outcomes. Table 3 presents the main possible predictive biomarkers of response to mitotane treatment.

### 6.1. Epigenetic Modifications

Epigenetic modifications refer to heritable changes in gene expression that do not involve changes to the underlying DNA sequence [83]. These modifications can profoundly impact cancer progression and drug resistance. In the context of mitotane resistance in ACC, several epigenetic mechanisms have been implicated, including DNA methylation, histone modifications, and microRNAs (miRNAs).

#### 6.1.1. DNA Methylation

DNA methylation involves the addition of a methyl group to the 5-carbon of cytosine residues, typically in CpG dinucleotides. Global hypomethylation can activate oncogenes, while the hypermethylation of specific CpG islands can silence tumor suppressor genes. The hypermethylation of the promoter regions of tumor suppressor genes (e.g., *RASSF1A*, *CDKN2A*) leads to their transcriptional silencing, contributing to unchecked cell proliferation and survival despite mitotane treatment. The hypomethylation of oncogenes and repetitive elements can lead to genomic instability and increased oncogenic signaling pathways, promoting resistance [84]. Among specific gene pathways affected by DNA methylation in ACC there is O6-Methylguanine-DNA Methyltransferase (*MGMT)*. The promoter hypermethylation of MGMT is associated with a favorable response to alkylating agents in some cancers but may play a complex role in ACC. Studies suggest that the hypermethylation and subsequent silencing of MGMT could be a mechanism of resistance or sensitivity, depending on the context and interaction with other pathways [83].

#### 6.1.2. Histone Modifications

There are many types of modifications: (1) Acetylation consists of the addition of acetyl groups to histone tails, generally associated with transcriptional activation. (2) Methylation is the addition of methyl groups, which can either activate or repress transcription depending on the context (e.g., H3K4me3 is activating, H3K27me3 is repressive). (3) Phosphorylation and Ubiquitination are other modifications that impact chromatin structure and gene expression. Histone Deacetylases (HDACs) remove acetyl groups, leading to the chromatin condensation and transcriptional repression of genes that might include tumor suppressors [85]. HDAC inhibitors are being investigated to counteract resistance by reactivating suppressed tumor suppressor genes and restoring sensitivity to mitotane [86]. Polycomb Repressive Complex 2 (PRC2) mediates the trimethylation of H3K27 (H3K27me3), leading to gene silencing [87,88]. The dysregulation of PRC2 components has been associated with cancer progression and resistance. The altered expression of PRC components has been linked to poor prognosis and resistance to conventional therapies, including mitotane.

#### 6.1.3. MicroRNAs (miRNAs)

miRNAs are small non-coding RNAs that regulate gene expression post-transcriptionally by binding to complementary sequences on target mRNAs, leading to their degradation or inhibition of translation [89]. OncomiRs: miRNAs that promote oncogenesis, such as miR-483-5p and miR-21, can inhibit tumor suppressor genes and promote survival pathways, contributing to resistance. Tumor Suppressor miRNAs: miRNAs such as let-7 and miR-34 that suppress oncogenes may be downregulated in resistant tumors, leading to enhanced proliferation and survival. There are several miRNAs whose expression is altered in ACC. The overexpression of miR-483-5p is associated with worse prognosis and resistance to mitotane, since it targets and downregulates pro-apoptotic genes, promoting cell survival [90]. miR-195 typically acts as a tumor suppressor. The downregulation of miR-195 in ACC is associated with increased cell proliferation and resistance [91]. miR-503 is another tumor suppressor miRNA and its reduced expression in ACC contributes to unchecked tumor growth and mitotane resistance [91]. Preclinical studies on ACC cell lines show that low miR-431 expression in Stage IV ACC is associated with resistance to adjuvant treatment. Zinc Finger E-Box Binding Homeobox 1 (ZEB1), a target of miR-431, plays a role in reversing epithelial–mesenchymal transition (EMT), enhancing the tumor’s response to therapies [92]. Additionally, miRNA signatures such as overexpressed miR-483-5p and miR-100-5p contribute to mitotane resistance by disrupting apoptosis and drug metabolism. Profiling miRNAs could reveal mechanisms of resistance and help identify novel therapeutic targets for ACC.

### 6.2. Steroidogenic Factor-1

Steroidogenic Factor 1 (*SF-1*), also known as *NR5A1*, is a critical transcription factor involved in the regulation of steroid hormone biosynthesis and the development of adrenal and gonadal tissues [62,64]. In ACC, SF-1 has emerged as a significant player not only in the biology of the disease but also as a predictive marker for treatment outcomes, particularly concerning mitotane resistance [93]. Elevated SF-1 levels are associated with increased cell proliferation, invasion, and steroidogenesis, contributing to the malignancy and clinical behavior of ACC. As a biomarker, SF-1 can be used to predict the likelihood of response to mitotane, guiding treatment decisions and allowing for more personalized therapeutic approaches. In primary resistance, where ACCs are inherently resistant to mitotane from the onset of treatment, high SF-1 levels are often observed. SF-1 mediated resistance can develop through different mechanisms. SF-1 regulates genes that encode enzymes involved in drug metabolism and efflux pumps, which can decrease intracellular concentrations of mitotane, thereby diminishing its cytotoxic effects [45]. SF-1 enhances the expression of anti-apoptotic proteins and survival pathways, such as BCL-2 and AKT, which can counteract the pro-apoptotic actions of mitotane. Finally, by upregulating steroidogenic enzymes, SF-1 can alter cellular metabolism in ways that promote cell survival and growth, further contributing to drug resistance [62]. In cases of acquired resistance, SF-1 may drive adaptive changes in gene expression upregulating genes involved in detoxification and cellular stress responses and inducing epigenetic changes that alter gene expression profiles, supporting a resistant phenotype. Developing inhibitors that specifically target SF-1’s transcriptional activity could potentially reverse resistance by downregulating key survival and metabolic pathways. Techniques such as RNA interference (RNAi) or CRISPR/Cas9-mediated gene editing could be employed to reduce SF-1 expression in ACC cells, thereby decreasing resistance mechanisms [94].

### 6.3. Insulin-like Growth Factor 2

Insulin-like Growth Factor 2 (IGF2) is a crucial growth factor involved in cell growth, development, and metabolism. IGF2 is often found to be overexpressed in ACC due to genetic and epigenetic alterations, such as the loss of imprinting or gene amplification [95,96]. This overexpression leads to increased cell proliferation, the inhibition of apoptosis, and enhanced metastatic potential. IGF2 promotes tumor growth through its interaction with the IGF1 receptor (IGF1R) and the insulin receptor (IR), triggering downstream signaling pathways that drive oncogenesis. The primary pathways activated by IGF2 include the PI3K/AKT, which promotes cell survival and growth by inhibiting apoptotic processes and facilitating cell cycle progression, and MAPK/ERK pathways, involved in cell division, differentiation and migration [95]. The overexpression of IGF2 can be used as a predictive biomarker for mitotane resistance, helping to stratify patients who may benefit from alternative or additional therapeutic approaches [95,96]. To overcome mitotane resistance associated with IGF2 overexpression, several therapeutic strategies are being explored: (1) IGF1R Inhibitors: Blocking the IGF1 receptor, which mediates the effects of IGF2, can disrupt downstream signaling pathways, and IGF1R inhibitors have shown promise in preclinical studies and early-phase clinical trials [31,97]; (2) PI3K/AKT/mTor Pathway Inhibitors [97]; (3) Combination Therapies: Combining mitotane with IGF1R inhibitors or PI3K/AKT inhibitors may provide a synergistic effect, improving treatment outcomes in ACC patients with high IGF2 expression [98,99].

### 6.4. CYP3A4

CYP3A4 is a crucial enzyme involved in the metabolism of numerous drugs, including mitotane. While primarily found in the liver, CYP3A4 is also present in other tissues, including the adrenal cortex. Its role in mitotane resistance in ACC hinges on its ability to metabolize the drug, thereby affecting both its efficacy and toxicity profile [100]. CYP3A4 can convert mitotane into inactive metabolites, reducing the drug’s effective concentration in the plasma and tumor tissue. This enhanced metabolism can lead to sub-therapeutic levels of mitotane, diminishing its cytotoxic effects on ACC cells. Research has shown that higher CYP3A4 expression levels correlate with increased mitotane metabolism, resulting in lower drug efficacy. Patients with elevated CYP3A4 activity may require higher doses of mitotane to achieve therapeutic levels, which can also increase the risk of side effects. Certain polymorphisms may lead to increased or decreased enzyme activity, impacting mitotane metabolism. Mitotane can induce the expression of CYP3A4, enhancing its own metabolism (autoinduction) as well as the metabolism of other drugs [67]. Clinical observations indicate that patients on long-term mitotane therapy often need dosage adjustments due to autoinduction. The co-administration of other CYP3A4 inducers or inhibitors can significantly alter mitotane pharmacokinetics, affecting its therapeutic efficacy [100]. CYP3A4 expression within tumor tissue itself can contribute to localized drug resistance. Tumors with high CYP3A4 expression can metabolize mitotane more effectively, protecting cancer cells from its cytotoxic effects. Immunohistochemical analyses of ACC samples have demonstrated variable CYP3A4 expression levels. Personalized dosing strategies can optimize drug levels, improving therapeutic outcomes while minimizing toxicity. Therapeutic drug monitoring (TDM) can be employed to measure plasma concentrations of mitotane and adjust doses accordingly to overcome rapid metabolism in patients with high CYP3A4 activity. Biomarker studies focusing on CYP3A4 mRNA levels or protein expression in tumor biopsies could aid in stratifying patients and predicting treatment outcomes [67]. Understanding the impact of CYP3A4 on mitotane pharmacokinetics and dynamics is crucial for optimizing treatment strategies.

### 6.5. CYP2W16 and CYP2B66 Single Nucleotide Polymorphisms (SNPs)

The cytochrome P450 (CYP) superfamily is vital for metabolizing endogenous and exogenous substances, including drugs. Genetic variations, such as single nucleotide polymorphisms (SNPs) in CYP genes, can reduce enzyme activity, affecting drug metabolism and potentially causing therapeutic failure or side effects. CYP2W1, primarily expressed in fetal tissues and certain tumors like colon cancer, is linked to poor prognosis but has unclear SNP-related functional impacts. In contrast, CYP2B6 and CYP3A4 are critical for mitotane metabolism. The CYP2B6*6 SNP, which lowers enzyme expression and activity, and CYP2W1*6 are associated with altered mitotane plasma levels, as shown in preclinical and clinical studies [101,102].

### 6.6. CXCL12 (SDF-1)

CXCL12, also known as stromal cell-derived factor 1 (SDF-1), is a chemokine that plays a critical role in various physiological processes, including stem cell homing, angiogenesis, and immune surveillance [103]. CXCL12 exerts its effects primarily through binding to its receptor, CXCR4, although it can also interact with CXCR7. CXCL12 plays a crucial role in angiogenesis by recruiting endothelial progenitor cells and promoting the formation of new blood vessels. The CXCL12/CXCR4 axis can modulate the immune microenvironment of tumors by attracting regulatory T cells (Tregs) and myeloid-derived suppressor cells (MDSCs), which suppress anti-tumor immune responses. This pathway can also activate downstream signaling cascades, such as the PI3K/AKT and MAPK pathways, which are involved in cell survival and drug resistance. CXCL12-mediated signaling enhances the expression of anti-apoptotic proteins and drug efflux pumps, contributing to reduced sensitivity to chemotherapeutic agents like mitotane [104]. Studies have shown that CXCL12 and CXCR4 are overexpressed in ACC tissues compared to normal adrenal tissues [103]. Immunohistochemical analyses reveal that high levels of CXCL12/CXCR4 are associated with increased tumor aggressiveness and metastatic potential [105]. Drugs that inhibit CXCR4, such as AMD3100 (plerixafor) and other small molecule antagonists, have shown potential in preclinical models to reduce ACC cell proliferation, migration, and invasion [106]. These agents can disrupt the CXCL12/CXCR4 signaling pathway, thereby inhibiting tumor growth and metastasis [107].

### 6.7. Circulating Fascin-1 (FSCN-1)

FSCN-1 is an actin-bundling protein that plays a crucial role in cell motility and structural integrity. It is involved in the formation of cellular protrusions like filopodia and invadopodia, which are essential for cell migration and invasion. High FSCN-1 expression has been linked to increased metastatic potential and poorer prognosis in several cancers. In ACC, studies have indicated that elevated levels of FSCN-1 in the bloodstream could reflect tumor activity and burden [108]. Monitoring these levels might provide insights into disease progression and response to mitotane therapy. Patients with elevated Fascin-1 levels tend to have a more aggressive disease course, characterized by higher rates of metastasis and lower overall survival. Given its role in promoting cell motility and invasion, FSCN-1 represents a potential therapeutic target [109]. Inhibiting Fascin-1 function could impair the invasive capabilities of ACC cells, potentially reducing metastasis and improving patient outcomes. FSCN-1 also affects the tumor microenvironment by influencing the behavior of stromal cells and the extracellular matrix. This can create a more favorable environment for tumor growth and metastasis. Clinical studies measuring circulating Fascin-1 levels in ACC patients have found correlations between high Fascin-1 levels and disease progression, suggesting its utility as a non-invasive biomarker for monitoring treatment response and disease status [109].

### 6.8. RRM1/RRM2

Ribonucleotide reductase (RR) is a crucial enzyme for DNA synthesis and repair, responsible for converting ribonucleotides into deoxyribonucleotides. It consists of two subunits: *RRM1* (Ribonucleotide Reductase M1) and *RRM2* (Ribonucleotide Reductase M2) [110]. The dysregulation of *RRM1* and *RRM2* has been implicated in cancer development, progression, and resistance to chemotherapy. Moreover, *RRM1* subunit is involved in the suppression of cell proliferation, cell migration, and metastases. *RRM1* and *RRM2* expressions can be regulated by oncogenic signaling pathways such as the *PI3K/AKT*/mTOR pathway [82]. These pathways are often activated in ACC, further enhancing the expression and activity of RRM1 and RRM2. In SW13 cells, mitotane specifically up-regulates RRM1, which hampers the conversion of mitotane into its active metabolites, consequently diminishing its anti-proliferative effects. The lower expression levels of RRM1 have been linked to longer recurrence-free survival in ACC patients undergoing adjuvant therapy. Additionally, the targeted silencing of RRM1 was shown to restore sensitivity to mitotane in SW13 ACC cells that were poorly responsive to the treatment, as demonstrated in vitro [111]. Knockdown experiments using siRNA against RRM1 or RRM2 in ACC cell lines have shown reduced cell proliferation, increased apoptosis, and heightened sensitivity to chemotherapeutic agents [112]. Given their role in DNA synthesis and repair, RRM1 and RRM2 are attractive targets for novel cancer therapies. The inhibitors of ribonucleotide reductase, such as hydroxyurea, have shown potential in preclinical models to reduce ACC cell viability and enhance the efficacy of other chemotherapeutic agents [113].

### 6.9. SOAT-1

Mitotane targets and inhibits SOAT-1 (ACAT-1), an enzyme critical for cholesterol esterification in the adrenal cortex. This inhibition causes toxic lipid accumulation and endoplasmic reticulum (ER) stress, leading to apoptosis in adrenocortical cells. Given its role, a SOAT-1 inhibitor has undergone a phase 1 clinical trial as a potential therapy for advanced ACC [114]. SOAT-1 is highly expressed in normal adrenal tissue, adrenocortical cell lines, and ACC, showing variability in tumor expression but minimal presence in non-adrenal tissues. In a study of 231 ACC patients treated with mitotane, SOAT-1 expression varied significantly between the adjuvant and the advanced disease groups [64]. Besides SOAT-1 inhibition, mitotane may also impair mitochondrial function, contributing to tissue-specific toxicity. Enhancing mitotane’s efficacy with SOAT-1 inhibitors could further disrupt lipid metabolism and amplify cytotoxic effects on ACC cells.

### 6.10. Estrogen-Related Receptors (ERR)

In the past decade, estrogen-related receptors (ERRs) and PPARγ-coactivator-1s (PGC-1s) have been shown to collaboratively regulate mitochondrial biogenesis and key metabolic pathways [115]. ERRα, a critical subtype, governs energy balance in both normal and diseased states. Identified as having cholesterol as its natural ligand, ERRα’s activity is enhanced through the recruitment of PGC-1s, boosting its transcriptional function [116,117]. The overexpression of ERRα has been linked to aggressive tumor behaviors in cancers like breast, ovarian, prostate, and colon, making it a potential prognostic marker for hormone-related tumors [118,119]. ERRα also influences tumor cell motility and metastasis, partly through metabolic reprogramming tied to processes like EMT [120]. In ACC, high ERRα expression correlates with poor mitotane response, as it may activate survival-promoting metabolic pathways [121].

### 6.11. Fetal and Adult Testis Expressed 1 (FATE-1)

Programmed cell death can be initiated by enhancing calcium (Ca^2+^) transfer from the endoplasmic reticulum (ER) to the mitochondria, primarily at contact points called mitochondria-associated membranes (MAMs) [122]. FATE-1, a cancer-testis antigen, regulates the spacing between the ER and mitochondria and controls mitochondrial Ca^2+^ uptake [123]. Encoded by a gene on Xq28.3, FATE1 is normally expressed in the testis and adrenal glands but is overexpressed in various cancers [124]. It is a 21-kDa protein found in MAMs and is related to the mitochondrial fission factor (Mff) protein family, though it lacks the domain necessary for fission processes [123]. Studies show that increased FATE1 expression reduces apoptosis and desensitizes cells to mitotane by impairing mitochondrial Ca^2+^-dependent pro-apoptotic signaling [125,126]. In patients with adrenocortical tumors receiving mitotane, those with higher tumor expressions of FATE1 exhibited worse outcomes compared to those with lower expression [122,125]. In ACC, higher FATE1 levels are linked to poorer survival outcomes and resistance to mitotane, underscoring its role in therapy resistance and disease progression [125,127].

### 6.12. Lipid Storage

Mitotane’s adrenocorticolytic effects are partly driven by lipotoxicity due to the buildup of intracellular free cholesterol (FC), primarily caused by its inhibition of cholesterol storage through the suppression of SOAT1 [40,64]. Lipid droplets (LDs), critical in cancer cell survival and metastasis, differ significantly between mitotane-sensitive (H295R) and resistant (MUC-1) ACC cell lines [40]. Sensitive cells predominantly store cholesteryl esters (CEs), while resistant cells mainly accumulate triacylglycerols (TAGs) [40]. This variation extends to lipid-handling proteins: sensitive cells express high levels of receptors like SCARB1 and LRP1 for cholesterol uptake and the lipolytic enzyme hormone-sensitive lipase (HSL), whereas resistant cells show the reduced expression of these proteins but higher levels of DGAT1, which promotes TAG storage [41,62].

Mitotane treatment in vitro induces a dose-dependent increase in HSL expression and a reduction in perilipins (PLIN1 and PLIN3), which regulate lipid droplet integrity [40,41,42,43,44]. This shift leads to decreased CE storage and increased FC levels, contributing to cell death in sensitive models. However, inhibiting lipolysis via HSL or ATGL inhibitors prevents mitotane-induced cell death, highlighting the role of lipid metabolism in treatment sensitivity. The distinct lipid-handling mechanisms in sensitive versus resistant ACC models suggest metabolic adaptation as a hallmark of drug resistance, providing a promising avenue for developing novel therapies for mitotane-resistant ACC.

### 6.13. ACSL4, ABCG2 (BCRP), and ABCB1 (P-gp, MDR1)

ATP-Binding Cassette Subfamily G Member 2 (ABCG2), also known as Breast Cancer Resistance Protein (BCRP), and ATP-Binding Cassette Subfamily B Member 1 (ABCB1) are members of the ATP-binding cassette (ABC) transporter superfamily, known for their role in drug efflux and multidrug resistance [128]. The *ABCB1* gene encodes for P-glycoprotein, a drug efflux pump involved in multidrug resistance. The overexpression of *ABCB1* has been associated with the reduced intracellular accumulation of mitotane and resistance to treatment in preclinical studies [66]. The elevated expression of ABCG2 and ABCB1 has been linked to resistance to multiple anti-cancer drugs, including mitotane. These transporters facilitate the efflux of mitotane from ACC cells, reducing its intracellular accumulation and cytotoxic effects. Preclinical studies have demonstrated that the inhibition or modulation of ABCG2 and ABCB1 can sensitize ACC cells to mitotane treatment [128]. ABCG2 and ABCB1 expression levels could serve as predictive biomarkers for mitotane response in ACC patients [66]. Acyl-CoA Synthetase Long-Chain Family Member 4 (ACSL4) is an enzyme involved in lipid metabolism, particularly in the biosynthesis of long-chain fatty acid-CoA esters [129]. It plays a crucial role in maintaining cellular membrane composition and integrity. Alterations in ACSL4 expression can impact lipid metabolism, cellular signaling, and drug resistance mechanisms. Studies have implicated ACSL4 in mediating resistance to various chemotherapeutic agents, including mitotane in ACC [128]. Increased ACSL4 expression has been associated with reduced sensitivity to mitotane-induced cytotoxicity. Preclinical models have demonstrated that ACSL4 inhibition sensitizes cancer cells to mitotane treatment, suggesting its role in mediating drug resistance [129].

### 6.14. Geminin (GMNN)

GMNN is a protein involved in regulating cell cycle progression and DNA replication. It acts as a crucial inhibitor of DNA replication licensing factor, preventing the re-replication of DNA during the cell cycle [130]. In normal cells, GMNN expression is tightly controlled, ensuring proper cell cycle progression and genomic stability. Immunohistochemical analyses reveal strong GMNN staining in ACC tumors, particularly in aggressive subtypes with high proliferative activity [131]. The GMNN-mediated dysregulation of DNA replication licensing may contribute to genomic instability and the accumulation of genetic alterations, driving tumor evolution and metastasis [132]. Animal models of ACC have shown that the manipulation of GMNN expression levels influences tumor growth and metastatic potential [131]. The overexpression of GMNN accelerates tumor progression, while the depletion of GMNN inhibits tumor growth and metastasis, underscoring its significance in ACC pathogenesis. High GMNN expression levels in ACC patients are associated with adverse clinical outcomes, including resistance to mitotane, shorter disease-free survival and overall survival. This suggests that GMNN may serve as a prognostic biomarker for predicting disease progression and therapeutic response. Small molecule inhibitors or nucleic acid-based approaches targeting GMNN could be developed to disrupt its oncogenic functions and impede tumor growth [130].

**Table 3:** Possible predictive biomarkers of response to mitotane treatment. DFS = disease free survival; OS = overall survival; DCR = disease control rate; TTP = time to progression; ChIP on chip = chromatin immunoprecipitations coupled to gene array technology; SAGE = serial analysis of gene expression.

Biomarker	Gene(s)/Locus	Function	Resistanece Mechanisms	Observation Methods	Clinical Outcomes	References
DNA Methylation	*RASSF1A*, *CDKN2A*, *MGMT*, others	Global hypomethylation can activate oncogenes, while hypermethylation of specific CpG islands can silence tumor suppressor genes	Hypomethylation of oncogenes can lead to genomic instability and increased oncogenic signaling pathways	RT-qPCR, Immuno-histochemistry	Poor mitotane response	[80,81,82,83,84]
Histone Modifications	*HDACs*, *PRC2*, others	Chromatin and transcriptional regulation	Transcriptional disregulation	ChIP on chip, SAGE	Poor prognosis and poor mitotane response	[84,85,86,87,88]
miRNAs	miR-483-5p, miR-21, miRlet-7, miR-34, miR-195	Binding to complementary sequences on target mRNAs, leading to their degradation or inhibition of translation	Disregulation in oncogene and tumor suppressor genes	RT-qPCR	Poor mitotane response	[89,90,91,92]
*SF-1*	SF-1 9q33.3	Transcription factor involved in the regulation of steroid hormone biosynthesis and the development of adrenal and gonadal tissues	Upregulation of steroidogenic enzymes, promotion of cell survival and growth	Immuno-histochemistry	Poor mitotane response	[45,62,64,93,94]
Insulin-Like Growth Factor 2	IGF-2 11p15.5	Involved in cell growth	Increased cell proliferation, inhibition of apoptosis, and enhanced metastatic potential	Immuno-histochemistry	Poor mitotane response	[95,96,97,98,99]
*CYP3A4*	CYP3A4 7q22.1	Involved in drugs metabolism	High CYP3A4 expression can metabolize mitotane more effectively, protecting cancer cells from its cytotoxic effects	Immuno-histochemistry	Low mitotane plasma levels	[67,100]
CYP2W1*6 CYP2B6*6 (SNPs)	CYP2W1 7p22.3 CYP2B6 19q13.2	Involved in drugs metabolism	Lower enzyme expression hampers the conversion of mitotane into its active metabolites	PCR	Low mitotane plasma levels, reduced DCR and TTP	[101,102]
*CXCL12* *(SDF-1)*	CXCL12 10q11.21	CXCL12/CXCR4 axis is involved in angiogenesis, immune response, and apoptosis	Inhibition of pro-apoptotic signaling and activation of pathways associated with cell proliferation and drug resistance	Immuno-histochemistry	Over-expression is associated with poor prognosis	[103,104,105,106,107]
*Fascin-1*	FSCN-1 7p22.1	Plays a role in the organization of actin filament bundles and the formation of microspikes, membrane ruffles, and stress fibers	Elevated Fascin-1 levels are associated with higher rates of metastasis	Western blot, qRT-PCR; Immuno- histochemistry	Poor prognosis, reduction in OS	[108,109]
*Rinocluotide Reductase*	RRM1/ RRM2 11p15.4/2p25.1	Converts ribonucleotides into deoxyribonucleotides	Higher expression hampers the conversion of mitotane into its active metabolites	qRT-PCR	Shorter DFS and OS	[110,111,112,113]
*SOAT-1*	SOAT-1 1q25.2	Sterol O-Acyltransferase 1 (SOAT-1)	Protein involved in steroidogenesis	Western blot, qRT-PCR; Immuno-histochemistry	SOAT1 expression translates into response to mitotane	[64]
*ERR*	ESRRA 11q13.1	Recruitment of PGC-1s, boosting its transcriptional function	Disregulation of mitochondrial biogenesis and key metabolic pathways	RNA analysis, Western blot	Poor mitotane response	[115,116,117,118,119,120,121]
*FATE-1*	FATE-1 Xq28	Regulates the spacing between the ER and mitochondria and controls mitochondrial Ca^2+^ uptake	Impairement in mitochondrial pro-apoptotic signaling. Overexpression is associated with increased steroidogenic and decreased immune response gene expression.	Immuno-histochemistry; Immunofluorescence, ELISA Western blot	FATE-1 expression seems to correlate with 5-year DFS and OS.	[122,123,124,125,126,127]
ACSL4	ACSL4 Xq23	Acyl-CoA Synthetase Long-Chain Family Member 4	Involved in lipid metabolism, reduction in mitotane induced cytotoxicity	Western blot, qRT-PCR; in H295R cell lines	Overexpression in highly aggressive, metastatic ACC-derived cells	[128,129]
ABCG2	ABCG2 4q22.1	BCRP, drug efflux pump	Reduced intracelullar accumulation of mitotane	Western blot, qRT-PCR; in H295R cell lines	Poor mitotane response	[66,128]
ABCB1 (P-gp, MDR1)	*ABCB1* 7q21.12	P-gp, drug efflux pump	Reduced intracellular accumulation of mitotane	Western blot, qRT-PCR; in H295R cell lines	Poor mitotane response	[66,128]
Geminin	*GMNN* 6p22.3	DNA replication Inhibitor	Genomic instability trough DNA-dysregulation	Immunohisto chemistry	Shorter DFS and OS in animal models	[130,131,132]

## 7. Conclusions

ACCs are rare and aggressive malignancies with limited therapeutic options and generally poor prognoses. Surgical intervention is currently the most effective approach, with the key goal of achieving clear surgical margins, or R0 resection. However, beyond surgery, the treatment landscape for ACC remains challenging, particularly in the context of systemic therapies.

Among the pharmacological agents available, mitotane has been the cornerstone of both adjuvant and metastatic treatment for many years. Yet, despite its long-standing use, mitotane remains a drug shrouded in uncertainty, particularly regarding its pharmacokinetics, pharmacodynamics, and the precise management of its side effects. This underscores the need for a deeper understanding of mitotane’s role in ACC treatment, especially as we consider its potential as a targeted therapy.

In this review, we aim to provide a broad overview of the existing literature, with a particular focus on identifying novel predictive and prognostic factors that could influence the response to mitotane. The intention is to present mitotane not merely as an old drug with historical significance but as a potential targeted therapy that could benefit from renewed clinical investigation.

One of the major challenges in ACC treatment is the lack of prospective data, as most of the prognostic scores we rely on have been derived from retrospective cohorts. This highlights a significant gap in our knowledge and an urgent need for well-designed clinical trials. Understanding the factors that predict a patient’s response to mitotane, whether in terms of primary or acquired resistance, could pave the way for more personalized treatment approaches.

Currently, the management of mitotane treatment is fraught with difficulties, not least because mitotanemia has not been proven to be a reliable predictor of treatment efficacy. Surprisingly, effective responses have been observed at serum concentrations below the traditionally accepted therapeutic range of 14–20 mg/L, which calls into question the utility of this marker and suggests that we need to look deeper into other potential indicators of treatment response.

This review highlights the importance of ongoing research into mitotane and ACC, as advancing our knowledge in this area could provide clinicians with greater confidence in using mitotane, despite its challenging pharmacologic profile. Identifying novel predictive and prognostic factors will be key to positioning mitotane as a viable targeted therapy and could pave the way for combination therapies with other anti-neoplastic agents. Such strategies have the potential to enhance treatment efficacy, prevent or overcome resistance, and ultimately improve patient outcomes.

In conclusion, while mitotane has long been a staple in ACC treatment, further well-conducted clinical trials are essential to fully establish its potential as a targeted therapy. The continued exploration of mitotane’s role in ACC, supported by advances in predictive and prognostic biomarkers, could lead to more personalized and effective treatment approaches for this challenging malignancy.

## Figures and Tables

**Table 1:** Main studies regarding mitotane treatment. We give priority to present prospective and randomized trials over the retrospective ones. RCT = Randomized Controlled Trial; pts = patients; PFS = Progression Free Survival; QoL = Quality of Life; RFS = Recurrence Free Survival; TTP = Time To Progression; ORR = Objective Response Rate; DOR = Duration Of Response; DFS = Disease Free Survival.

Trial Name	Trial Design	Authors and Year of Publication	Population	Investigated Schemes	Outcomes	References
*FIRM-ACT*	Phase III RCT Open Label	Fassnacht M, Terzolo M et al. 2012	304 pts with Advanced ACC	EDP-Mitotane vs. STZ-Mitotane	OS (primary); PFS, Tumor Response, QoL (secondary)	[26]
ADIUVO	Phase III RCT Open Label	Terzolo M, Fassnacht M et al. 2023	91 pts with R0, stage I–III, Ki67 ≤ 10% ACC	Adjuvant Mitotane for 2 years vs. Surveillance	OS (primary); RFS (secondary)	[24]
ADIUVO-2	Phase III RCT	Mouhammed A Habra Last Update October 2024, actually ongoing	240 pts with High Risk ACC (Stage I–III, R0 relapsed within 90 days, R1, Rx, Ki67 > 10%)	Adjuvant EDP × four cycles plus Mitotane for 2 years vs. Adjuvant Mitotane for 2 years	RFS (primary); OS, Safety, QoL (secondary)	[25]
Gemcitabine plus metronomic 5-fluorouracil or capecitabine as a second-/third-line chemotherapy in advanced ACC: a multicenter phase II study	Multicenter Phase II Study	Sperone P, Ferrero A et al. 2010	28 pts whit II/III line advanced ACC	Mitotane plus Gemcitabine plus metronomic 5-FU or capecitabine	PFS (primary); Tumor Response Rate, TTP, OS (secondary)	[27]
Mitotane Monotherapy in Patients With Advanced Adrenocortical Carcinoma	Multicenter cohort study	Megerle F, Herrmann W et al. 2018	127 pts with advanced ACC	Mitotane montherapy	PFS, OS	[29]
cabACC trial	Single-arm Phase II trial	Laganà M, Grisanti S et al. 2022	25 pts with II/III line advanced ACC	Cabazitaxel plus Mitotane (only in pts with secretory ACC)	PFS (primary); ORR, OS, hormone response in secretory ACC (secondary)	[30]
NCT00778817	Phase I/II trial	Lerario AM, Worden FP et. Al 2014	2p chemiotherapy naïve pts with recurrent/metastatic ACC	Mitotane plus IMC-A12 vs. Mitotane	Efficay and Safety (phase I) PFS (phase II)	[31]
Comparison of two mitotane starting dose regimens in patients with advanced adrenocortical carcinoma	Prospective, open-label, multicenter trial	Kerkhofs TM, Baudin E, Terzolo M et al. 2013	40 mitotane-naïve pts with metastatic ACC	Low vs. High Mitotane dose regimen	The difference in median mitotane plasma levels between both treatment groups	[32]
High-dose mitotane strategy in adrenocortical carcinoma: prospective analysis of plasma mitotane measurement during the first 3 months of follow-up	Single-center, prospective study	Mauclère-Denost S, Leboulleux S et al. 2011	22 pts with ACC	High-dose (4g/day or more) vs. Low-dose Mitotane regimens	Percentage of patients who achieve a plasma mitotane level above 14 mg/L; Safety within the first 3 months of treatment	[33]
Etoposide, doxorubicin and cisplatin plus mitotane in the treatment of advanced adrenocortical carcinoma: a large prospective phase II trial	Multicenter Phase II trial	Berruti A, Terzolo M et al. 2005	72 pts with non resectable advanced ACC	EDP for maximun six cycles plus Mitotane	ORR (primary) TTP, OS (secondary)	[34]
Mitotane associated with etoposide, doxorubicin, and cisplatin in the treatment of advanced adrenocortical carcinoma. Italian Group for the Study of Adrenal Cancer	Multicenter Phase II trial	Berruti A, Terzolo M et al. 1998	28 pts with non resectable advanced ACC	EDO plus Mitotane	ORR, TTP; hormone response	[35]
Plasma concentrations of o,p′DDD, o,p′DDA, and o,p′DDE as predictors of tumor response to mitotane in adrenocortical carcinoma: results of a retrospective ENS@T multicenter study	Multicenter Study	Hermsen IG, Fassnacht M et al. 2011	Blood Samples from 91 pts with diagnosis of advanced ACC	Mitotane	Correlation of the currently used mitotane threshold of 14 mg/L with tumor response	[36]
Phase II trial of mitotane and cisplatin in patients with adrenal carcinoma: a Southwest Oncology Group study	Phase II trial	Bukowski RM, Wolfe M et al. 1993	37 pts with residual or metastatic ACC stratified by risk	CDPP 100 mg/m^2^ or 75 mg/m^2^ plus Mitotane	ORR, DOR	[37]
Streptozocin and o,p′DDD in the treatment of adrenocortical cancer patients: long-term survival in its adjuvant use	Phase II trial	Khan TS, Imam H et al. 2000	40 pts with ACC	Mitotane plus STZ	DFS, OS	[38]
Gemcitabine-Based Chemotherapy in Adrenocortical Carcinoma: A Multicenter Study of Efficacy and Predictive Factors	Phase II trial	Henning JEK, Deutschbein T et al. 2017	145 pts with advanced ACC	Mitotane plus Gemcitabine plus capecitabine	ORR, PFS	[39]

**Table 2:** Most important targets of mitotane.

Gene	Locus	Protein	Protein Function	Effect of Mitotane	Cell Material	References
*STaR*	8p11.2	STAR, mitochondrial	Protein involved in steroidogenesis and cholesterol transfer across the mitochondrial membrane	Inhibition	H295R	[60]
*CYP11A1*	15q24.1	Cholesterol side-chain cleavage enzyme, (CYP11A1)	Protein involved in steroidogenesis	Inhibition	NCI-H295, NCI-H295R	[61]
*HSD3B2*	1p12	3 beta-hydroxysteroid dehydrogenase/Delta 5-->4-isomerase type 2 (HSD3B2)	Protein involved in steroidogenesis	Inhibition	NCI-H295, NCI-H295R	[61]
*CYP21A2*	6p21.33	Steroid 21-hydroxylase (CYP21A2)	Protein involved in steroidogenesis	Inhibition	NCI-H295, NCI-H295R	[61]
*CYP11B1*	8q24.3	Cytochrome P450 11B1, 11-beta-hydroxylase, (CYP11B1)	Protein involved in steroidogenesis	Inhibition	NCI-H295, NCI-H295R	[45,61]
*CYP17A1*	10q24.32	Steroid 17-alpha-hydroxylase/17,20 lyase (CYP17A1)	Protein involved in steroidogenesis	Inhibition	NCI-H295, NCI-H295R	[61]
*ABCG1*	21q22.3	ATP-binding cassette sub-family G member 1	Involved in macrophage cholesterol and phospholipids transport	Inhibition		[66]
*SCARB1*	12q24.31	Scavenger receptor class B type 1	Receptor for high density lipoprotein	Inhibition		[64]
*SOAT-1*	1q25.2	Sterol O-Acyltransferase 1 (SOAT-1)	Protein involved in steroidogenesis	Inhibition	H295R, SW13	[64]
Complex I (43 subunits)		NADH–ubiquinone oxidoreductase complex	Respiratory chain Complex I	Inhibition	H295R	[45]
Complex IV (14 subunits)		Cytochrome *c* oxidase	Respiratory chain Complex IV	Inhibition	H295R	[45]
*Bax*	19q13.33	BCL2-associated X protein (Bax)	Apoptosis regulator	Induction	H295R	[45,53]
*Bcl-2*	18q21.33	BCL-2 family	Apoptosis regulator	Inhibition	H295R	[45,53]
*CYP3A4*	7q22.1	CYP3A4	Hepatic cytochrome involved in metabolism of various drugs, including anti-hypertensives, statins, anti-biotics, chemotherapeutic agents, and coumarin-like anti-coagulants	Induction	HepG2	[67]
*NR5A1*	9q33.3	Steroidogenic Factor-1 (SF-1)	Transcriptional regulator for an array of different genes related to sex determination and differentiation, reproduction, and metabolism	Inhibition	H295R, H295A	[62,68]
*SHBG*	17p13.1	Sex Hormones Binding Globulin (SHBG)	Hormones carrier through bloodstream	Induction increased expression of SHBG mRNA	HepG2, Hep89	[69]
*CBG* (SerpinA, Transcortin)	14q32.13	Corticosteroid Binding Blobulin (CGB)	Hormones carrier through bloodstream	Induction increased expression of CBG mRNA	HepG2, Hep89	[69]
Proteomic Profile		D-3-PGDH, triose phosphate isomerase and α-enolase, adrenodoxin reductase	Proteins involved in energetic metabolism and electron flow through Respiratpry chain	Modulation	H295R	[70]
Proteomic Profile		*HSP70*, *Prx II Prx VI*, *HSP27*, *HSP71A*	Proteins involved in stress response	Induction (Prx II, IV and HSP71A) Inhibition (HSP27, HSP70)	H295R	[70,71]
Proteomic Profile		tubulin-β isoform II, profilin-1	Proteins involved in cytoskeleton	Modulation	H295R	[70]
Proteomic Profile		Hint, PHB, hnRNP, cathepsin D	Proteins involved in tumorigenesis, mithocondrial development, and aging	Modulation	H295R	[70,72,73]
